# HIV-1 Gag C-terminal amino acid substitutions emerging under selective pressure of protease inhibitors in patient populations infected with different HIV-1 subtypes

**DOI:** 10.1186/s12977-014-0079-7

**Published:** 2014-09-25

**Authors:** Guangdi Li, Jens Verheyen, Kristof Theys, Supinya Piampongsant, Kristel Van Laethem, Anne-Mieke Vandamme

**Affiliations:** Clinical and Epidemiological Virology, Rega Institute for Medical Research, Department of Microbiology and Immunology, KU Leuven - University of Leuven, Leuven, Belgium; Institute of Virology, University Hospital, University Duisburg-Essen, Essen, Germany; Centro de Malária e Outras Doenças Tropicais and Unidade de Microbiologia, Instituto de Higiene e Medicina Tropical, Universidade Nova de Lisboa, Lisboa, Portugal

**Keywords:** HIV-1 subtype, Protease, Protease inhibitor, Gag amino acid substitution, Drug resistance

## Abstract

HIV-1 Gag amino acid substitutions associated with protease inhibitor (PI) treatment have mainly been reported in subtype B, while information on other subtypes is scarce. Using sequences from 11613 patients infected with different HIV-1 subtypes, we evaluated the prevalence of 93 Gag amino acid substitutions and their association with genotypic PI resistance. A significant association was found for 13 Gag substitutions, including A431V in both subtype B and CRF01_AE. K415R in subtype C and S451G in subtype B were newly identified. Most PI-associated Gag substitutions are located in the flexible C-terminal domain, revealing the key role this region plays in PI resistance.

**Electronic supplementary material:**

The online version of this article (doi:10.1186/s12977-014-0079-7) contains supplementary material, which is available to authorized users.

## Findings

An amino acid substitution is commonly defined as an amino acid change between two consecutive sequences based on longitudinal data [1,2]. Amino acid substitutions in HIV-1 protease, commonly called resistance mutations if they confer HIV-1 drug resistance, are known to emerge under selective pressure of protease inhibitors (PIs) [3]. As an alternative mechanism, HIV-1 can escape PI selective pressure by the selection of substitutions in the protease substrate Gag [1,4-7]. Such Gag substitutions arising during PI-based treatment have mostly been characterized in HIV-1 subtype B (Additional file 1: Table S1), while only a few studies have focused on non-B subtypes using small cohorts of patients (Table 1). Gag variability has been shown to impact PI susceptibility in a subtype-dependent manner [4,6], warranting a comprehensive analysis of PI-associated Gag substitutions across different subtypes. Here, we identified novel Gag substitutions in HIV-1 non-B subtypes using longitudinal data from patients failing PI-based therapy. Moreover, we evaluated the prevalence of the newly identified and the previously reported Gag substitutions in different HIV-1 subtypes and investigated their association with genotypic PI resistance using a large sequence dataset.

**Table 1:** **Summary of Gag amino acid substitutions in HIV-1 non-B subtypes observed during PI-based treatment**

Gag amino acid substitutions in non-B subtypes*	**Number of patients infected by non-B subtypes**	**Reference**
**A431V**	A or F^#^ (n = 4)	[8]
**K436R**, **N451S**	C (n = 1)	[8]
L363F, A364G, **A374T**, I376V, M378V, R380K, **K436E**, G443R	G (n = 2), 01_AE (n = 1), 02_AG (n = 4)	[9]
V128A/T/I, Q130R, **Y132F**, V135M, V362I, A373T, **A374T**, A375T, I376A/V/M, K380R, S381G, N382K, E428D/Q, Q430R/G/V/I, **A431V, K436R**, L449I, **N451S**, **R452K**, P453I	G (n = 21)	[10]
N375S, G381R	A1 (n = 2)	[2]
G381S, G446E	02_AG (n = 1)	[2]
V135I, I376V, L486F	01_AE (n = 1)	[2]
**P453L/T**/I	F^#^ (n = 61)	[11]
M138L, F363L, L363W, **A374T**, V374A, R387K, N389T, K411Q, K415R, G420A, P422Q, T427P, P445L, S451G, R452G, **P453L**, P453Ins, I469T, P472S, P474L, E477Q	A1 (n = 1), C (n = 6), D (n = 1), F1 (n = 1), J (n = 1), 01_AE (n = 1), 02_AG (n = 1)	Our study

**Figure 1:**
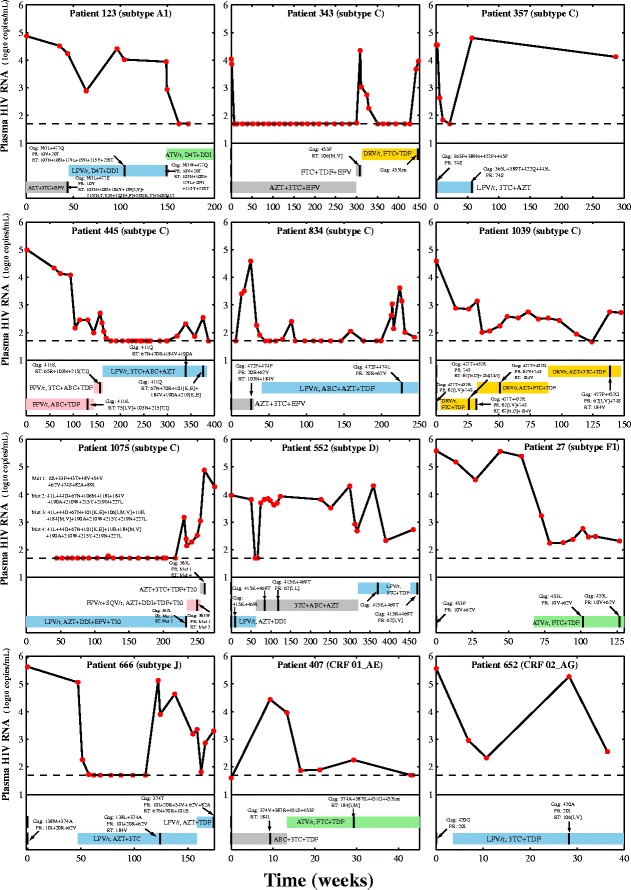
**Gag substitutions and PI or RTI resistance mutations in 12 patients from the Leuven cohort.** Each subplot shows the data of one patient regarding the viral load, the treatment period and the emerging Gag substitutions and the PI/RTI resistance mutations. X- and Y-axes indicate the time (weeks) and the level of plasma HIV RNA (log_10_ copies/mL), respectively. For each subplot, red dots indicate the level of viral load and the dash line indicates the viral load cutoff at 50 copies per mL. Beneath the viral load plot, each treatment period is annotated by a colored bar with vertical black lines indicating the sequence sampling time. The blue, pink, green and yellow bars show PI-based treatments containing LPV/r, FPV/r, ATV/r and DRV/r, respectively. The grey bar indicates treatments lacking PIs. Multiple substitutions or mutations are shown using the plus symbol “+”. Amino acids translated from ambiguous nucleotide letters are indicated by brackets. For patient 343, the insertion EPTAPP at position P453 is annotated as P453Ins. For patient 1075, the sets of PI or RTI resistance mutation are abbreviated (Mut 1-4) and listed in the subplot. Additional file 1: Table S2 provides the full list of Gag, protease and RT substitutions in these 12 patients.

*Non-B Gag substitutions reported during PI-based treatment. The substitutions are summarized based on the original publications, and for the substitutions in our study, it is given relative to the baseline sequences sampled from individual patients (see Figure 1). The substitutions also identified in subtype B are indicated in bold. Additional file 1: Table S1 summarizes the information of Gag substitutions in HIV-1 subtype B.

^#^Information of HIV-1 subtype or sub-subtype was ambiguous or not available.

We first investigated the emergence of non-B Gag substitutions during PI-based treatment in a cohort of 1068 patients followed at the University Hospital of Leuven, for which virological outcome and treatment information were available [12]. Our protocol and quality control of viral sequencing and viral load tests have been described previously [13,14]. For 69 patients infected with HIV-1 non-B subtypes and receiving PI-based treatment for at least three months, sequence information for Gag, protease and reverse transcriptase (RT) was available at baseline and at treatment failure, which was defined according to the guidelines of the European AIDS Clinical Society (EACS) (http://www.eacsociety.org/). Under drug selective pressure, 21 different substitutions at 18 Gag positions were identified among 12 patients, of whom 11 harbored Gag substitutions in the presence of (pre-existing or simultaneously acquired) drug resistance mutations in protease or RT (Figure 1, Additional file 1: Table S2). Gag substitution P453Ins (insertion: EPTAPP) emerged in patient 343 in the absence of PI and RTI resistance mutations. Some substitutions were from a less to a more common amino acid such as M138L. Specifically, patients failing LPV/r-based regimens developed one of the following Gag substitution patterns: L363W + E477Q, F363L + N389T + P422Q + P455L, K411Q, P472S + P474L, K415R + I469T, M138L, A374T or G420A. Patients failing DRV/r-based regimens developed Gag substitution patterns P453Ins or T427P + R452G. Patients failing an ATV/r-based regimen developed Gag substitution patterns: P453L or V374A + R387K + S451G + P453Ins. A patient failing a regimen containing FPV/r and SQV/r developed L363W. Longitudinal data from 34 PI-naïve patients infected with non-B subtypes revealed the emergence of one Gag substitution (V370A) in a single patient. Overall, when analyzing all subtypes, the proportion of PI-treated patients with Gag substitutions was much higher than that of PI-naïve patients (17.4% (12/69) vs 2.9% (1/34), p-value = 0.037).

**Figure 2:**
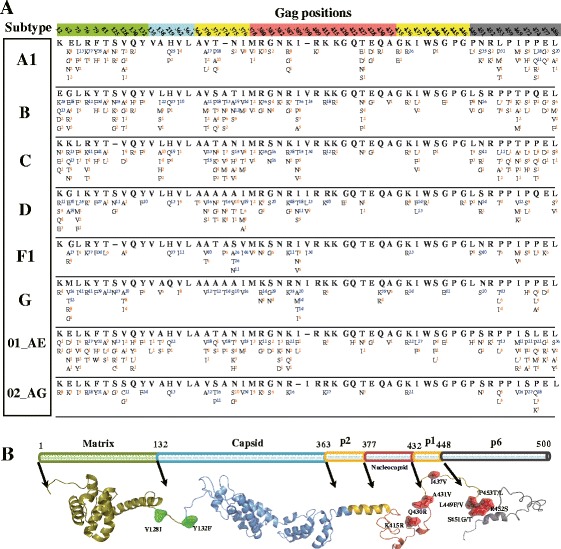
**Prevalence of Gag amino acid variants reported in patients failing PI-based therapies and their mapping to HIV-1 protein structures. (A)** Prevalence of amino acid variations at 55 Gag positions in 8 HIV-1 subtypes (A1, B, C, D, F1, G, 01_AE and 02_AG) given the Los Alamos full-length Gag sequence dataset (Table 2). Only Gag positions where amino acid substitutions have been observed during PI-based treatment are shown. For each position, the HXB2 index is shown at the top, followed by the most prevalent amino acids (bold) and amino acid variations in our sequence datasets. Amino acids with blue superscripts have prevalence above 10% and other amino acids have orange superscripts. **(B)** Structural representation of Gag polyprotein and mapping of the 13 PI-associated Gag substitutions identified in Table 3. The annotation of Gag polyproteins is shown at the top. Individual Gag protein structures are shown at the bottom. Gag substitutions are annotated and colored accordingly. Red surfaces indicate PI-associated Gag substitutions at the Gag C-terminal domain; other substitutions are shown in green. PDB data of Gag protein structures: matrix, 1HIW; capsid, 3NTE; p2, 1U57; nucleocapsid, 2M3Z; p6, 2C55. Visualization software: PyMOL V1.5 (http://www.pymol.org/).

For our second analysis, we compiled a comprehensive list of 93 Gag substitutions at 55 positions in B and non-B subtypes observed in PI-treated patients, based on literature results or our first analysis as described above (Table 1, Additional file 1: Table S1). Next, we systematically evaluated the prevalence of these variants in major HIV-1 subtypes using 10865 full-length Gag sequences retrieved from the HIV Los Alamos database (one sequence per patient) (Table 2). Sequence alignment and quality control have been described previously [15]. We found that the prevalence of 62 (66.7%) Gag variants at 39 positions was above 1% in at least one subtype or CRF (A1, B, C, D, F1, G, CRF01_AE, CRF02_AG). Among the 55 Gag positions, only 363 and 455 were highly conserved with less than 1% overall amino acid variation in every subtype and CRF in our dataset (Figure 2A). Moreover, 77 of these 93 variants (82.8%) were found at 42 positions located in the Gag C-terminal domain (positions: 362-500).

**Table 2:** **Summary of Leuven and Los Alamos sequence datasets**

**Subtype**	**Los Alamos dataset**	**Leuven dataset**			**Total number of patient**
	Number of Gag sequence*	**Number of Gag sequence**	**Number of PI-naïve patient**	**Number of PI-treated patient**	
A1	1648	167	72	19	1739
B	4131	639	313	57	4501
C	2780	198	58	24	2862
D	443	42	20	9	472
F1	35	38	25	4	64
G	49	1	1	0	50
J	3	8	1	2	6
01_AE	1714	72	45	5	1764
02_AG	62	139	71	22	155
Total	10865	1304	606	142	11613

*Number of Gag sequences in different HIV-1 subtypes (one sequence per patient) [15].

As treatment information of the 10865 full-length *gag* nucleotide sequences was largely lacking, our third analysis aimed to evaluate whether these 93 Gag variants were significantly associated with genotypic PI resistance. Among the 11613 sequences pooled from the Leuven and the Los Alamos datasets (Table 2), 6645 spanned both the *gag* and the full-length protease regions, and were translated into amino acid sequences for our analysis. Using the drug resistance interpretation algorithms HIVdb V7.0 [16] and Rega V9.1 [17], 660 sequences were concordantly estimated to be partially or fully resistant to at least one PI, and 5657 sequences were concordantly estimated to be fully susceptible to all PIs (Additional file 1: Table S3). Sequences with discordant estimates of PI susceptibility were excluded from our analysis. Fisher’s exact tests were then used to compare the amino acid prevalence between these PI-susceptible and PI-resistant datasets. Of the 93 Gag variants, 16 at 13 amino acid positions were associated with (partial or full) PI resistance in at least one HIV-1 subtype (p-value < 0.05, Additional file 1: Table S4). After multiple testing correction using the false discovery rate approach described in [18], 13 Gag variants at 10 positions remained significantly PI-associated within individual subtypes (adjusted p-value < 0.05), including 11 variants located in the Gag C-terminal domain (Figure 2B, Table 3). Our analysis successfully identified the known PI-associated Gag substitution A431V, strengthening the validity of our approach. As the only PI-associated Gag substitution found in more than one subtype, A431V had a high prevalence in the PI-resistant strains of subtype B (13.5%) and CRF01_AE (18.2%) (Table 3). Interestingly, of the 21 Gag substitutions observed in our first analysis, K415R and S451G were newly identified to be significantly associated with genotypic PI resistance in subtypes C and B respectively, suggesting a possible involvement in PI-resistance.

**Table 3:** **Prevalence of PI-associated Gag substitutions in individual HIV-1 subtypes**

Gag substitutions*	**Subtype**	**Prevalence** ^**#**^		**P-value**	**Adjusted p-value**
		**PI-resistant dataset**	**PI-susceptible dataset**		
V128I	B	5.8% (7/121&)	0.9% (6/638)	0.002	0.024
Y132F	B	10.7% (13/122)	3.4% (22/639)	0.004	0.035
K415R	C	2.5% (3/119)	0.0% (0/1727)	<0.0001	0.012
Q430R	C	2.5% (3/119)	0.1% (1/1727)	0.003	0.046
A431V	B	13.5% (23/170)	0.1% (1/787)	<0.0001	<0.0001
	01_AE	18.2% (4/22)	0.7% (8/1111)	<0.0001	0.007
I437V	B	8.9% (15/168)	1.7% (13/784)	<0.0001	<0.0001
L449F	B	5.6% (21/377)	0.5% (7/1352)	<0.0001	<0.0001
L449V	B	4.8% (18/377)	0.9% (12/1352)	<0.0001	<0.0001
S451G	B	3.4% (13/378)	1.3% (17/1348)	0.008	0.041
S451T	B	2.1% (8/378)	0.0% (0/1348)	<0.0001	<0.0001
R452S	B	3.4% (13/384)	0.3% (4/1374)	<0.0001	<0.0001
P453T	C	21.8% (26/119)	3.1% (53/1722)	<0.0001	<0.0001
P453L	B	18.5% (71/384)	7.1% (99/1399)	<0.0001	<0.0001

*A list of Gag substitutions whose prevalence differs significantly between sequences estimated to be (fully or partially) PI-resistant and sequences estimated to be PI-susceptible (see full reports in Additional file 1: Table S4). The substitutions are indicated relative to the consensus amino acids from individual subtypes [15]. One-tailed Fisher’s exact tests were performed, and p-values were adjusted using multiple testing correction via the false discovery rate (FDR) approach [18].

^#^Statistical analyses were only performed on individual subtype (B, C, G, 01_AE) datasets, which contained more than 10 (partially or fully) PI-resistant sequences. Additional file 1: Table S3 summarizes the subtype distribution of PI-resistant and PI-susceptible sequence datasets.

&: The numerator indicates the number of sequences for which the corresponding Gag position is covered; the denominator indicates the number of sequences displaying the respective amino acid substitutions.

To our knowledge, this study presents the first large-scale sequence analysis to establish statistical significance of PI-associated Gag substitutions in HIV-1 non-B subtypes. Our longitudinal analysis of a clinical cohort of patients failing PI-based therapy confirmed that PI-treated patients developed more Gag substitutions than PI-naïve patients. The majority of these Gag substitutions emerged in the context of pre-existing or simultaneously acquired PI or RTI resistance mutations, confirming the important role of the known resistance mutations, while in some patients Gag substitutions emerged in the absence of resistance mutations (Figure 1, Additional file 1: Table S2). Such Gag substitutions may therefore contribute to the virological failure of PI-based treatments. Based on two widely used genotypic interpretation algorithms, our comparative analysis found that only 13 (13.8%) of the 93 Gag substitutions emerging under PI selective pressure were significantly associated with genotypic PI resistance (Table 3). Particularly, the novel Gag substitutions K415R and S451G were identified in both our longitudinal and cross-sectional sequence analyses. This suggests that they may play a role in viral escape from PI selective pressure, partially contributing to the observed virological failure. Since virological outcome and treatment information is lacking for most sequences extracted from the HIV Los Alamos database, this limits our analysis to address the clinical impact of the newly identified substitutions with large-scale data. Using small cohorts, previous studies suggested that different subtypes may develop different Gag substitutions [6,19,20]. We confirmed this hypothesis since only 9 of the 58 Gag substitutions reported in non-B subtypes (Table 1) were also observed in subtype B (Additional file 1: Table S1). Among non-B Gag substitutions, 4 were significantly associated with genotypic PI resistance, of which only A431V was PI-associated in subtype B as well (Table 3). However, further evaluations on subtypes A2, D, F2, J, K and other CRFs are still needed due to the restriction of our study to particular subtypes. Interestingly, a predominant presence of PI-associated Gag substitutions at the flexible C-terminal domain of Gag (Figure 2B) leads us to suggest the hypothesis that PI-associated Gag substitutions tend to emerge in the structural flexible regions. These Gag substitutions can emerge along with protease drug resistance mutations as shown in our longitudinal sequence analysis (Figure 1, Additional file 1: Table S2) and previous studies [21,22]. Future studies are still needed to investigate the significance of coevolution between Gag substitutions and protease resistance mutations.

Overall, our findings showed different PI-associated substitutions in the Gag C-terminal domain across different subtypes, providing a roadmap to elucidate the role of Gag amino acid substitutions in the development of PI resistance.

Our Leuven sequences with associated information are available through Euresist (http://www.euresist.org). The protocol and this consent procedure have been approved by the Ethical Committee UZ Leuven (reference ML-8627, approval B322201316521 S52637). Our toolbox designed for visualizing the longitudinal data in Figure 1 is freely available in Additional file 2: Toolbox S1.

## Additional files

Additional file 1: Table S1. Summary of HIV-1 subtype B Gag amino acid substitutions observed during PI-based treatment. **Table S2.** Summary of Gag, protease and RT amino acid substitutions in the Leuven cohort. **Table S3.** Summary of PI-resistant and PI-susceptible sequence datasets. **Table S4.** Prevalence of Gag amino acid variants in individual HIV-1 subtypes. Additional file 2: Software. Toolbox S1: Our Matlab toolbox designed for visualizing longitudinal data of viral load, treatment period and sampling time.

## Abbreviations

CRF: Circulating recombinant form
EACS: European AIDS clinical society
FDR: False discovery rate
PDB: Protein data bank
PR: Protease
PI: Protease inhibitor
RT: Reverse transcriptase
RTI: Reverse transcriptase inhibitor
