# Supplementary material for: HIV-1 Gag C-terminal amino acid substitutions emerging under selective pressure of protease inhibitors in patient populations infected with different HIV-1 subtypes
Source: Retrovirology. 2014 Sep 25;11:79. doi: 10.1186/s12977-014-0079-7 (PMC4189171; doi:10.1186/s12977-014-0079-7)
Supplement: Additional file 1: Table S1. — Summary of HIV-1 subtype B Gag amino acid substitutions observed during PI-based treatment. Table S2. Summary of Gag, protease and RT amino acid substitutions in the Leuven cohort. Table S3. Summary of PI-resistant and PI-susceptible sequence datasets. Table S4. Prevalence of Gag amino acid variants in individual HIV-1 subtypes. [file 12977_2014_79_MOESM1_ESM.pdf]

Supplementary tables

Table S1: Summary of HIV-1 subtype B Gag substitutions observed during PI-based treatment.

Gag protein	Gag position	Gag substitutions *	Reference
Matrix	12	E12K	[1]
Matrix	62	G62R	[2]
Matrix	75	L75R	[1]
Matrix	76	R76K	[3]
Matrix	79	Y79F	[4]
Matrix	81	T81A	[4]
Matrix	125	S125K	[2]
Matrix	132	Y132F	[2]
Capsid	219	H219Q	[1] [5]
p2	370	V370A, V370I, V370M	[6]
p2	373	S373Q	[7] [8]
p2	374	A374G,A374N,A374P,A374S,A374T	[7] [9]
p2	375	T375N	[7]
Nucleocapsid	390	V390D	[1]
Nucleocapsid	409	R409K	[1]
Nucleocapsid	431	A431V	[8] [10] [11] [12] [13] [14] [15] [16] [17] [18] [19]
p1	435	G435E, G435R	[12] [20] [21]
p1	436	K436E, K436N, K436R	[9] [12] [13] [20] [22]
p1	437	I437A, I437T, I437V	[9] [12] [13] [16] [19] [20] [22] [23] [24]
p1	438	W438R	[12]
p1	440	S440C	[20]
p6	449	L449F, L449P, L449Q, L449V	[8] [9] [10] [13] [16] [19] [20] [24] [25] [26] [27] [28] [29] [30]
p6	451	S451I, S451N, S451T	[30] [31]
p6	452	R452K, R452S	[10] [13] [19] [32]
p6	453	P453L, P453T	[9] [11] [13] [16] [17] [19] [26] [27] [29] [32]
p6	459	P459I	[33]

*: Only Gag substitutions associated with FDA-approved PIs in HIV-1 subtype B are summarized. The substitution is expressed relative to the subtype B consensus sequence (http://www.hiv.lanl.gov/).

Table S2: Summary of Gag, protease and RT substitutions in 12 patients of the Leuven cohort. For the first sequence, PI/RTI drug resistance mutations, detected by the drug resistance interpretation algorithms HIVdb v7.0 [34] and/or Rega V9.1 [35], are colored red. The other protease and RT variants are indicated in black. Ambiguous nucleotide letters are decomposed and translated into amino acids, which are indicated by brackets. Gag substitutions and PI/RTI resistance mutations with therapy changes are mapped in Figure 1. "na" indicates that the sequence does not cover the corresponding position.

Patient ID	Subtype	Sampling day	Gag substitution	Protease variants and PI resistance mutations	RT variants and RTI resistance mutations
123	A1	2002-10-08	363L+477E	10V	49R+103N+108I+118V+173[L,S]+179I+184V +189[I,V]+215[Y,N,T,S]+225[P,H]+238[T,K] +248T+303L
		2003-12-10	363L+477Q	10V+20T	49[R,K]+103N+108I+118V+173L+179L+184M +189I+215Y+225P+238T+248T+303[L,W]
		2004-10-27	363W+477Q	10V+20T	49K+103N+108I+118[V,I]+173L+179L+184M +189I+215Y+225P+ 238T+248[T,I]+303L
343	C	2010-03-17	453P	64I+70[K,R]	40[E,D]+49[K,R]+106[V,M]+123[S,G]+126[K,R] +165[I,T]+175N+277[K,R]+281R
		2012-11-30	453PTAPPE	64[I,L]+70K	40E+49K+106V+123S+126K+165I+175[N,Y] +277K+281[R,K]
357	C	2004-07-26	363F+389N +422P+445P	74S	13[K,R]+86D+148V+154[K,R]+280[C,Y]
		2005-09-05	363L+389T +422Q+445L	74S	13K+86[D,G]+148[V,I]+154K+280C
445	C	2007-06-27	411K	60[D,E]+66I	3S+28K+32E+36A+49K+60I+65K+67D+70K +75[V,I]+101K+103N+123[N,S]+173V+177D +184M+190G+207[E,G]+211R+214L+215[T,I]+ 219K+277[R,K]+ 278Q+281R
		2008-01-02	411K	60E+66I	3S+28K+32E+36A+49K+60I+65R+67D+70K +75V+101K+103N+123S+173V+177D+184M

					+190G+207E+211R+214L+215[T,I]+219K +277R+278[Q,R]+281K
		2011-07-15	411Q	60D+66[I,M]	3S+28E+32K+36E+49[K,R]+60V+65K+67N +70R+75V+101K+103K+123S+173A+177E +184V+190A+207E+211K+214[L,F]+215T +219K+277R+278Q+281K
		2012-04-04	411Q	60D+66I	3[N,S,D,G]+28[K,E]+32K+36E+49K+60V+65K +67N+70R+75V+101[K,E]+103K+123S+173A +177E+184V+190A+207E+211K+214[L,F] +215T+219[K,E]+277R+278Q+281K
834	C	2003-02-12	472P+474P	20R+62V	103N+173T +184V
		2007-01-04	472S+474L	20R+62V	103K+173A +184M
1039	C	2010-08-12	427T +452R	62[I,V]+72I+74S+82[I,V]	8[V,I]+28E+39E+48[T,S]+53[E,D]+67D+121[D,H] +123[S,N,D,G]+135I+142[I,V]+166[K,R]+184M +208H+214F+241V+286[A,T]+324[E,D] +334Q
		2011-02-14	427T +452R	62I+72I+ 74S+82V	8V+28E+39E+48T+53E+67[N,D]+121D+123S +135I+142[I,V]+166[K,R]+184[I,M]+208H+214F +241V+286A+324E+334[Q,H]
		2011-04-01	427T +452R	62[I,V]+72I+74S+82[I,V]	8V+28E+39E+48[T,S]+53[E,D]+67[N,D]+121D +123S+135I+142I+166[K,R]+184V+208[H,Y] +214F+241V+286A+324E+334na
		2011-08-08	427T +452G	62V+72I+74S+82I	8V+28K+39E+48T+53E+67D+121D+123S+135L +142I+166K+184V+208H+214F+241V+286A +324E+334Q
		2013-04-22	427P+452G	62[I,V]+72[I,M]+74S+ 82[I,V]	8V+28E+39[E,K]+48T+53E+67D+121D+123S +135[I,T,S,L]+142I+166K+184V+208H+214[F,L] +241[V,L]+286A+324E+334Q
1075	C	2005-11-07	363L	10I+18H+33F+43T+48V +54A+62V+74S+82A+89I	35T+41L+44D+67N+84[T,S]+101K+106M+ 118I+184V+190A+210W+215Y+219N+227L
		2006-03-01	363W	10I+18H+33F+43T+48V +54A+62V+74S+82A+89I	35T +41L+44D+67N+84T+101[K,E]+106[M,I,V] +118I+184[V,M]+190A+210W+215Y+219N+227L
		2006-05-15	363L	10I+18[H,Q]+33F+43T+48V +54A+62V+74S+82A+89I	35[T,I]+41L+44D+67N+84T+101[K,E]+106V+118I +184[V,M]+190A+210W+215Y+219N+227L
552	D	2002-02-18	415K+469I	13I+14K+62I+72I+93[I,L]	20[K,R]+136N+275K+294P+297A
		2003-07-28	415K+469T	13I+14K+62I+72I+93L	20[K,R]+136N+275[K,R]+294P+297A+334R+335G
		2004-03-22	415K+469T	13I+14K+62[I,V]+72[I,V] +93L	20[K,R]+136N+275K+294P+297A +334R+335E

		2009-02-11	415K+469T	13[I,V]+14[K,R]+62I +72[I,V]+93[I,L]	20K+136N+275K+294T+297[A,T]+334R+335E
		2011-01-24	415R+469T	13[I,V]+14[K,R]+62[I,V] +72[I,V]+93[I,L]	20K+136[N,H]+275K+294na+297na+334na+335na
27	F1	2008-07-24	453P	10V+62V+69Y	207E+276V
		2010-07-14	453L	10V+62V+69Y	207K+276[I,V]
		2011-01-12	453L	10V+62V+69[H,Y]	207[K,E]+276V
666	J	2001-06-13	138M+374A	10I+20R+35E+54I+60[S,N] +62V+82V+89M	67D+70K+101K+174[K,R]+184M+200I+288A +294T+322T
		2003-11-12	138L+374A	10I+20R+35E+54I+ 60S +62V+82V+89M	67D+70K+101K+174[K,R]+184V+200[I,T, A,V] +288[A,S]+294T+322T
		2004-11-03	374T	10I+20R+35D+54V+ 60S+62V+82A+89L	67N+70R+101E+174K+184M+200I+288A+294P +322A
407	01_AE	2009-11-02	374V+387R +451S+453P	79P	11K+184I+200A+281R+286A+304[E,A]+325L+326I
		2010-03-22	374A+387K +451G+ 453EPTAPP	79H	11[K,T]+184[I,M]+200[A,V]+281K+286[A,T] +304A+325I+326V
652	02_AG	2008-01-31	420G	20I	11R+27[T,S]+35I+106V+207G+276I+291D+292I +294T+311R
		2008-08-19	420A	20I	11[R,K,Q]+27T+35[I,T]+106[V,I]+207[E,G]+276[I,V] +291[D,E]+292[I,V]+294[T,P]+311[R,K]

Table S3: Summary of PI-resistant and PI-susceptible sequence datasets.

**Subtype/ CRF**	**Number of PI-susceptible Gag-protease sequences ***	**Number of PI-resistant Gag-protease sequences #**	**Total**
A1	185+72=257	6+3=9	266
B	1820+313=2133	434+31=465	2598
C	1728+58=1786	119+18=137	1923
D	98+20=118	1+0=1	119
F1	21+25=46	3+0=3	49
G	33+0=33	14+0=14	47
01_AE	1112+45=1157	22+2=24	1181
02_AG	55+71=126	3+4=7	133
Total	5657	660	6317

*: Number of PI-susceptible Gag-protease sequences used in this study. These sequences were amino acid sequences translated from nucleotide sequences obtained from the Los Alamos + Leuven datasets. PI-susceptible sequences were estimated to be fully susceptible to all PIs by both the HIVdb v7.0 [34] and the Rega V9.1 [35] algorithms.

#: Number of PI-resistant Gag-protease sequences used in this study. These sequences were amino acid sequences translated from nucleotide sequences obtained from the Los Alamos + Leuven datasets. PI-resistant sequences were estimated to be partially or fully resistant to at least one PI by both the HIVdb v7.0 [34] and the Rega V9.1 [35] algorithms. All the Los Alamos sequences encode the full-length Gag polyprotein.

Table S4: Prevalence in individual HIV-1 subtypes of Gag amino acid variants observed during PI therapy.

Gag amino acid variant	Subtype*	Amino acid prevalence		p-value #	Adjusted p-value
		PI-resistant dataset	PI-susceptible dataset		
12E	C	0.8%(1/119)	0.2%(4/1724)	0.286	1
12K	B	25.4%(31/122)	25.3%(158/624)	0.923	1
62G	C	1.7%(2/117)	0.2%(4/1700)	0.054	0.741
62R	B	4.1%(5/121)	4.1%(26/639)	0.599	1
76K	C	48.3%(57/118)	44.7%(769/1719)	0.999	1
76R	01_AE	9.1%(2/22)	19.1%(212/1112)	0.958	1
79F	G	14.3%(2/14)	48.5%(16/33)	0.998	1
79Y	01_AE	9.1%(2/22)	33.7%(374/1111)	0.999	1
81A	B	15.6%(19/122)	7.5%(48/639)	0.021	0.165
81A	01_AE	4.5%(1/22)	10.9%(121/1112)	0.929	1
125K	B	4.1%(5/122)	1.6%(10/636)	0.087	0.559
128A	B	0.8%(1/121)	2.8%(18/638)	0.966	1
128A	G	15.4%(2/13)	9.7%(3/31)	0.532	1
128I	B	5.8%(7/121)	0.9%(6/638)	0.002	0.024
128I	C	7.7%(9/117)	11.8%(201/1709)	0.964	1
128I	G	15.4%(2/13)	0.0%(0/31)	0.101	1
128I	01_AE	4.5%(1/22)	0.7%(8/1089)	0.172	1
130R	B	2.5%(3/122)	1.3%(8/639)	0.263	0.925
130R	G	7.1%(1/14)	0.0%(0/33)	0.313	1
132F	B	10.7%(13/122)	3.4%(22/639)	0.004	0.035
132F	G	21.4%(3/14)	0.0%(0/33)	0.035	0.925
135I	B	1.6%(2/122)	0.3%(2/657)	0.121	0.625
135I	C	0.8%(1/119)	0.2%(3/1728)	0.236	1
135M	B	0.8%(1/122)	0.0%(0/657)	0.158	0.694
135M	G	7.1%(1/14)	0.0%(0/32)	0.319	1
138M	B	10.7%(13/122)	8.4%(55/657)	0.369	1
138M	C	0.8%(1/119)	0.2%(3/1721)	0.236	1
219Q	B	28.1%(34/121)	21.2%(141/665)	0.458	1
219Q	C	9.2%(11/119)	18.3%(314/1715)	0.999	1
219Q	01_AE	22.7%(5/22)	22.8%(253/1112)	0.768	1
362I	B	17.7%(22/124)	21.9%(149/680)	0.978	1
362I	C	2.5%(3/119)	1.6%(27/1727)	0.317	1
362I	G	21.4%(3/14)	0.0%(0/33)	0.035	0.925
363F	B	0.8%(1/124)	0.0%(0/680)	0.155	0.694
364G	C	0.8%(1/119)	0.1%(1/1728)	0.126	1
370A	B	17.8%(23/129)	14.7%(106/720)	0.491	1
370I	B	3.9%(5/129)	2.4%(17/720)	0.254	0.925
370M	B	4.7%(6/129)	6.1%(44/720)	0.83	1
370V	01_AE	36.4%(8/22)	31.3%(348/1111)	0.768	1
373A	B	4.9%(7/143)	4.8%(37/766)	0.6	1
373Q	B	0.7%(1/143)	2.0%(15/766)	0.938	1
373S	C	5.9%(2/34)	2.4%(27/1114)	0.229	1

373T	B	3.5%(5/143)	4.0%(31/766)	0.719	1
374G	B	2.8%(4/142)	1.1%(8/756)	0.114	0.625
374G	C	1.5%(1/65)	0.2%(1/591)	0.191	1
374N	B	8.5%(12/142)	5.4%(41/756)	0.168	0.706
374P	B	2.8%(4/142)	5.2%(39/756)	0.938	1
374S	B	1.4%(2/142)	3.0%(23/756)	0.929	1
374T	B	13.4%(19/142)	14.2%(107/756)	0.819	1
374V	B	0.7%(1/142)	2.5%(19/756)	0.97	1
374V	C	9.2%(6/65)	14.7%(87/591)	0.955	1
375A	B	28.0%(40/143)	17.2%(131/761)	0.089	0.559
375A	C	1.8%(2/111)	2.2%(34/1513)	0.727	1
375N	B	15.4%(22/143)	19.2%(146/761)	0.973	1
375N	B	15.4%(22/143)	19.2%(146/761)	0.973	1
375S	B	6.3%(9/143)	11.4%(87/761)	0.989	1
375T	C	0.9%(1/111)	0.5%(7/1513)	0.436	1
375T	G	14.3%(2/14)	0.0%(0/22)	0.171	1
376A	B	0.7%(1/144)	0.3%(2/763)	0.407	1
376A	G	15.4%(2/13)	3.0%(1/33)	0.227	1
376M	B	3.5%(5/144)	2.1%(16/763)	0.249	0.925
376M	C	2.5%(3/118)	0.3%(5/1724)	0.012	0.251
376M	G	7.7%(1/13)	3.0%(1/33)	0.512	1
376V	B	15.3%(22/144)	18.0%(137/763)	0.945	1
376V	C	9.3%(11/118)	6.8%(118/1724)	0.282	1
376V	G	23.1%(3/13)	12.1%(4/33)	0.412	1
378V	B	1.3%(2/153)	1.0%(8/769)	0.52	1
378V	C	0.8%(1/119)	0.9%(15/1726)	0.66	1
380K	B	29.4%(45/153)	25.0%(192/769)	0.771	1
380K	01_AE	4.5%(1/22)	10.5%(117/1111)	0.922	1
380R	G	7.1%(1/14)	15.2%(5/33)	0.91	1
381G	C	22.7%(27/119)	18.1%(310/1714)	0.488	1
381G	G	28.6%(4/14)	24.2%(8/33)	0.688	1
381S	B	2.6%(4/153)	2.3%(18/770)	0.527	1
382K	B	1.3%(2/153)	1.2%(9/768)	0.575	1
382K	G	7.1%(1/14)	0.0%(0/33)	0.313	1
387K	B	4.6%(7/152)	5.5%(42/769)	0.764	1
387R	01_AE	27.3%(6/22)	20.5%(223/1090)	0.526	1
389N	B	2.7%(4/150)	4.2%(32/758)	0.884	1
389N	C	4.4%(5/114)	2.1%(35/1682)	0.12	1
389T	C	9.6%(11/114)	17.8%(300/1682)	0.998	1
389T	G	8.3%(1/12)	0.0%(0/2)	0.867	1
415R	B	0.6%(1/169)	1.7%(13/786)	0.937	1
415R	C	2.5%(3/119)	0.0%(0/1727)	<0.0001	0.012
427P	B	1.2%(2/168)	0.6%(5/784)	0.363	1
427P	C	0.8%(1/119)	0.0%(0/1725)	0.065	0.771
430R	C	2.5%(3/119)	0.1%(1/1727)	0.003	0.046
430R	G	15.4%(2/13)	0.0%(0/33)	0.093	1
431V	B	13.5%(23/170)	0.1%(1/787)	<0.0001	<0.0001
431V	C	1.7%(2/119)	0.2%(4/1728)	0.054	0.741
431V	G	23.1%(3/13)	0.0%(0/33)	0.03	0.925

431V	01_AE	18.2%(4/22)	0.7%(8/1111)	<0.0001	0.007
435E	B	0.6%(1/170)	0.0%(0/785)	0.179	0.715
436R	B	4.7%(8/170)	4.8%(38/787)	0.637	1
436R	C	3.4%(4/119)	4.1%(71/1724)	0.75	1
436R	G	15.4%(2/13)	15.2%(5/33)	0.716	1
436R	01_AE	31.8%(7/22)	27.0%(300/1112)	0.702	1
437V	B	8.9%(15/168)	1.7%(13/784)	<0.0001	<0.0001
437V	C	1.7%(2/119)	0.6%(11/1726)	0.208	1
438R	B	0.6%(1/170)	0.3%(2/787)	0.446	1
449F	B	5.6%(21/377)	0.5%(7/1352)	<0.0001	<0.0001
449I	B	1.9%(7/377)	1.0%(13/1352)	0.132	0.644
449P	B	6.9%(26/377)	8.0%(108/1352)	0.871	1
449P	C	2.5%(3/119)	3.0%(52/1727)	0.716	1
449V	B	4.8%(18/377)	0.9%(12/1352)	<0.0001	<0.0001
451G	B	3.4%(13/378)	1.3%(17/1348)	0.008	0.041
451N	B	13.0%(49/378)	14.7%(198/1348)	0.962	1
451S	C	42.0%(50/119)	38.5%(663/1721)	0.992	1
451S	G	14.3%(2/14)	6.1%(2/33)	0.395	1
451T	B	2.1%(8/378)	0.0%(0/1348)	<0.0001	<0.0001
452G	B	0.8%(3/384)	0.1%(2/1374)	0.074	0.542
452G	G	7.7%(1/13)	0.0%(0/33)	0.298	1
452K	B	1.0%(4/384)	0.9%(13/1374)	0.535	1
452S	B	3.4%(13/384)	0.3%(4/1374)	<0.0001	<0.0001
453I	B	0.8%(3/384)	0.2%(3/1399)	0.12	0.625
453I	G	15.4%(2/13)	0.0%(0/33)	0.093	1
453Ins	C	25.7%(35/136)	24.0%(413/1722)	0.3561	1
453L	B	18.5%(71/384)	7.1%(99/1399)	<0.0001	<0.0001
453L	C	3.4%(4/119)	10.4%(179/1722)	0.999	1
453T	B	4.4%(17/384)	4.7%(66/1399)	0.699	1
453T	C	21.8%(26/119)	3.1%(53/1722)	<0.0001	<0.0001
453T	01_AE	40.9%(9/22)	14.1%(157/1112)	0.026	0.386
469I	B	3.1%(12/383)	2.9%(46/1570)	0.508	1
469I	C	1.7%(2/116)	1.1%(19/1660)	0.411	1
469T	G	14.3%(2/14)	0.0%(0/33)	0.102	1
472P	01_AE	9.5%(2/21)	13.1%(121/927)	0.821	1
472S	B	0.9%(4/426)	1.6%(28/1795)	0.893	1
472S	C	1.8%(2/114)	7.6%(119/1560)	0.999	1
474L	B	0.5%(2/430)	0.3%(6/1811)	0.475	1
474P	B	0.7%(3/430)	1.7%(30/1811)	0.968	1
474P	01_AE	4.5%(1/22)	21.2%(218/1028)	0.996	1
486F	B	0.2%(1/433)	0.5%(9/1806)	0.884	1
486F	C	0.8%(1/119)	0.1%(1/1728)	0.126	1

*: We only examined the sequence datasets of subtypes B, C, G and CRF01_AE, which contained more than 10 sequences estimated to be (partially or fully) PI-resistant (see Table S3).

#: One-tailed Fisher's exact tests were performed on amino acid variants that occurred more than once in individual subtype datasets. For each HIV-1 subtype, the obtained p-values were adjusted using multiple testing corrections via the false discovery rate approach (software: Matlab 2013a, also see reference [36]). Amino acid variants are colored orange if their p-values and adjusted p-values were lower than 0.05. Amino acid variants are colored blue if their p-values were lower than 0.05 but adjusted p-values were above 0.05. Amino acid variants whose p-values are equal to 1 are not shown.

References

1. Aoki M, Venzon DJ, Koh Y, Aoki-Ogata H, Miyakawa T, Yoshimura K, Maeda K, Mitsuya H: **Non-cleavage site gag mutations in amprenavir-resistant human immunodeficiency virus type 1 (HIV-1) predispose HIV-1 to rapid acquisition of amprenavir resistance but delay development of resistance to other protease inhibitors.** *J Virol* 2009, **83:**3059-3068.
2. Chang MW, Oliveira G, Yuan J, Okulicz JF, Levy S, Torbett BE: **Rapid deep sequencing of patient-derived HIV with ion semiconductor technology.** *J Virol Methods* 2013, **189:**232-234.
3. Fun A, Wensing AM, Verheyen J, Nijhuis M: **Human Immunodeficiency Virus Gag and protease: partners in resistance.** *Retrovirology* 2012, **9:**63.
4. Parry CM, Kolli M, Myers RE, Cane PA, Schiffer C, Pillay D: **Three residues in HIV-1 matrix contribute to protease inhibitor susceptibility and replication capacity.** *Antimicrob Agents Chemother* 2011, **55:**1106-1113.
5. Gatanaga H, Suzuki Y, Tsang H, Yoshimura K, Kavlick MF, Nagashima K, Gorelick RJ, Mardy S, Tang C, Summers MF, Mitsuya H: **Amino acid substitutions in Gag protein at non-cleavage sites are indispensable for the development of a high multitude of HIV-1 resistance against protease inhibitors.** *J Biol Chem* 2002, **277:**5952-5961.
6. Seclen E, Gonzalez Mdel M, Corral A, de Mendoza C, Soriano V, Poveda E: **High prevalence of natural polymorphisms in Gag (CA-SP1) associated with reduced response to Bevirimat, an HIV-1 maturation inhibitor.** *AIDS* 2010, **24:**467-469.
7. Larrouy L, Charpentier C, Landman R, Capitant C, Chazallon C, Yeni P, Peytavin G, Damond F, Brun-Vezinet F, Descamps D, group As: **Dynamics of gag-pol minority viral populations in naive HIV-1-infected patients failing protease inhibitor regimen.** *AIDS* 2011, **25:**2143-2148.
8. Malet I, Roquebert B, Dalban C, Wirden M, Amellal B, Agher R, Simon A, Katlama C, Costagliola D, Calvez V, Marcelin AG: **Association of Gag cleavage

sites to protease mutations and to virological response in HIV-1 treated patients. *J Infect* 2007, **54:**367-374.
9. Ghosn J, Delaugerre C, Flandre P, Galimand J, Cohen-Codar I, Raffi F, Delfraissy JF, Rouzioux C, Chaix ML: **Polymorphism in Gag gene cleavage sites of HIV-1 non-B subtype and virological outcome of a first-line lopinavir/ritonavir single drug regimen.** *PLoS One* 2011, **6:**e24798.
10. Mo H, Parkin N, Stewart KD, Lu L, Dekhtyar T, Kempf DJ, Molla A: **Identification and structural characterization of I84C and I84A mutations that are associated with high-level resistance to human immunodeficiency virus protease inhibitors and impair viral replication.** *Antimicrob Agents Chemother* 2007, **51:**732-735.
11. Nijhuis M, Wensing AM, Bierman WF, de Jong D, Kagan R, Fun A, Jaspers CA, Schurink KA, van Agtmael MA, Boucher CA: **Failure of treatment with first-line lopinavir boosted with ritonavir can be explained by novel resistance pathways with protease mutation 76V.** *J Infect Dis* 2009, **200:**698-709.
12. van Maarseveen NM, Andersson D, Lepsik M, Fun A, Schipper PJ, de Jong D, Boucher CA, Nijhuis M: **Modulation of HIV-1 Gag NC/p1 cleavage efficiency affects protease inhibitor resistance and viral replicative capacity.** *Retrovirology* 2012, **9:**29.
13. Kolli M, Stawiski E, Chappey C, Schiffer CA: **Human immunodeficiency virus type 1 protease-correlated cleavage site mutations enhance inhibitor resistance.** *J Virol* 2009, **83:**11027-11042.
14. Larrouy L, Lambert-Niclot S, Charpentier C, Fourati S, Visseaux B, Soulie C, Wirden M, Katlama C, Yeni P, Brun-Vezinet F, et al: **Positive impact of HIV-1 gag cleavage site mutations on the virological response to darunavir boosted with ritonavir.** *Antimicrob Agents Chemother* 2011, **55:**1754-1757.
15. Knops E, Kemper I, Schulter E, Pfister H, Kaiser R, Verheyen J: **The evolution of protease mutation 76V is associated with protease mutation 46I and gag mutation 431V.** *AIDS* 2010, **24:**779-781.
16. Banke S, Lillemark MR, Gerstoft J, Obel N, Jorgensen LB: **Positive selection pressure introduces secondary mutations at Gag cleavage sites in human immunodeficiency virus type 1 harboring major protease resistance mutations.** *J Virol* 2009, **83:**8916-8924.
17. Bally F, Martinez R, Peters S, Sudre P, Telenti A: **Polymorphism of HIV type 1 gag p7/p1 and p1/p6 cleavage sites: clinical significance and implications for resistance to protease inhibitors.** *AIDS Res Hum Retroviruses* 2000, **16:**1209-1213.
18. Cote HC, Brumme ZL, Harrigan PR: **Human immunodeficiency virus type 1 protease cleavage site mutations associated with protease inhibitor cross-resistance selected by indinavir, ritonavir, and/or saquinavir.** *J Virol* 2001, **75:**589-594.
19. Verheyen J, Litau E, Sing T, Daumer M, Balduin M, Oette M, Fatkenheuer G, Rockstroh JK, Schuldenzucker U, Hoffmann D, et al: **Compensatory mutations at the HIV cleavage sites p7/p1 and p1/p6-gag in therapy-naive and therapy-experienced patients.** *Antivir Ther* 2006, **11:**879-887.
20. Knops E, Brakier-Gingras L, Schulter E, Pfister H, Kaiser R, Verheyen J:

Mutational patterns in the frameshift-regulating site of HIV-1 selected by protease inhibitors. *Med Microbiol Immunol* 2012, **201:**213-218.

21. Larrouy L, Vivot A, Charpentier C, Benard A, Visseaux B, Damond F, Matheron S, Chene G, Brun-Vezinet F, Descamps D, Cohort ACH-: **Impact of gag genetic determinants on virological outcome to boosted lopinavir-containing regimen in HIV-2-infected patients.** *AIDS* 2013, **27:**69-80.
22. Nijhuis M, van Maarseveen NM, Lastere S, Schipper P, Coakley E, Glass B, Rovenska M, de Jong D, Chappey C, Goedegebuure IW, et al: **A novel substrate-based HIV-1 protease inhibitor drug resistance mechanism.** *PLoS Med* 2007, **4:**e36.
23. Lambert-Niclot S, Flandre P, Malet I, Canestri A, Soulie C, Tubiana R, Brunet C, Wirden M, Katlama C, Calvez V, Marcelin AG: **Impact of gag mutations on selection of darunavir resistance mutations in HIV-1 protease.** *J Antimicrob Chemother* 2008, **62:**905-908.
24. Larrouy L, Chazallon C, Landman R, Capitant C, Peytavin G, Collin G, Charpentier C, Storto A, Pialoux G, Katlama C, et al: **Gag mutations can impact virological response to dual-boosted protease inhibitor combinations in antiretroviral-naive HIV-infected patients.** *Antimicrob Agents Chemother* 2010, **54:**2910-2919.
25. Prado JG, Wrin T, Beauchaine J, Ruiz L, Petropoulos CJ, Frost SD, Clotet B, D'Aquila RT, Martinez-Picado J: **Amprenavir-resistant HIV-1 exhibits lopinavir cross-resistance and reduced replication capacity.** *AIDS* 2002, **16:**1009-1017.
26. Brann TW, Dewar RL, Jiang MK, Shah A, Nagashima K, Metcalf JA, Falloon J, Lane HC, Imamichi T: **Functional correlation between a novel amino acid insertion at codon 19 in the protease of human immunodeficiency virus type 1 and polymorphism in the p1/p6 Gag cleavage site in drug resistance and replication fitness.** *J Virol* 2006, **80:**6136-6145.
27. Maguire MF, Guinea R, Griffin P, Macmanus S, Elston RC, Wolfram J, Richards N, Hanlon MH, Porter DJ, Wrin T, et al: **Changes in human immunodeficiency virus type 1 Gag at positions L449 and P453 are linked to I50V protease mutants in vivo and cause reduction of sensitivity to amprenavir and improved viral fitness in vitro.** *J Virol* 2002, **76:**7398-7406.
28. Myint L, Matsuda M, Matsuda Z, Yokomaku Y, Chiba T, Okano A, Yamada K, Sugiura W: **Gag non-cleavage site mutations contribute to full recovery of viral fitness in protease inhibitor-resistant human immunodeficiency virus type 1.** *Antimicrob Agents Chemother* 2004, **48:**444-452.
29. Roquebert B, Malet I, Wirden M, Tubiana R, Valantin MA, Simon A, Katlama C, Peytavin G, Calvez V, Marcelin AG: **Role of HIV-1 minority populations on resistance mutational pattern evolution and susceptibility to protease inhibitors.** *AIDS* 2006, **20:**287-289.
30. Kolli M, Lastere S, Schiffer CA: **Co-evolution of nelfinavir-resistant HIV-1 protease and the p1-p6 substrate.** *Virology* 2006, **347:**405-409.
31. Kaufmann GR, Suzuki K, Cunningham P, Mukaide M, Kondo M, Imai M, Zaunders J, Cooper DA: **Impact of HIV type 1 protease, reverse transcriptase, cleavage site, and p6 mutations on the virological response to quadruple**

therapy with saquinavir, ritonavir, and two nucleoside analogs. *AIDS Res Hum Retroviruses* 2001, **17:**487-497.

32. Yates PJ, Hazen R, St Clair M, Boone L, Tisdale M, Elston RC: **In vitro development of resistance to human immunodeficiency virus protease inhibitor GW640385.** *Antimicrob Agents Chemother* 2006, **50:**1092-1095.
33. Lastere S, Dalban C, Collin G, Descamps D, Girard PM, Clavel F, Costagliola D, Brun-Vezinet F, Group NT: **Impact of insertions in the HIV-1 p6 PTAPP region on the virological response to amprenavir.** *Antivir Ther* 2004, **9:**221-227.
34. Liu TF, Shafer RW: **Web resources for HIV type 1 genotypic-resistance test interpretation.** *Clinical infectious diseases* 2006, **42:**1608-1618.
35. Van Laethem K, De Luca A, Antinori A, Cingolani A, Perna CF, Vandamme AM: **A genotypic drug resistance interpretation algorithm that significantly predicts therapy response in HIV-1-infected patients.** *Antivir Ther* 2002, **7:**123-129.
36. Storey JD: **A direct approach to false discovery rates.** *Journal of the Royal Statistical Society: Series B (Statistical Methodology)* 2002, **64:**479-498.
