# Supplementary figures and images for: HIV-1 Gag C-terminal amino acid substitutions emerging under selective pressure of protease inhibitors in patient populations infected with different HIV-1 subtypes
Source: Retrovirology. 2014 Sep 25;11:79. doi: 10.1186/s12977-014-0079-7 (PMC4189171; doi:10.1186/s12977-014-0079-7)

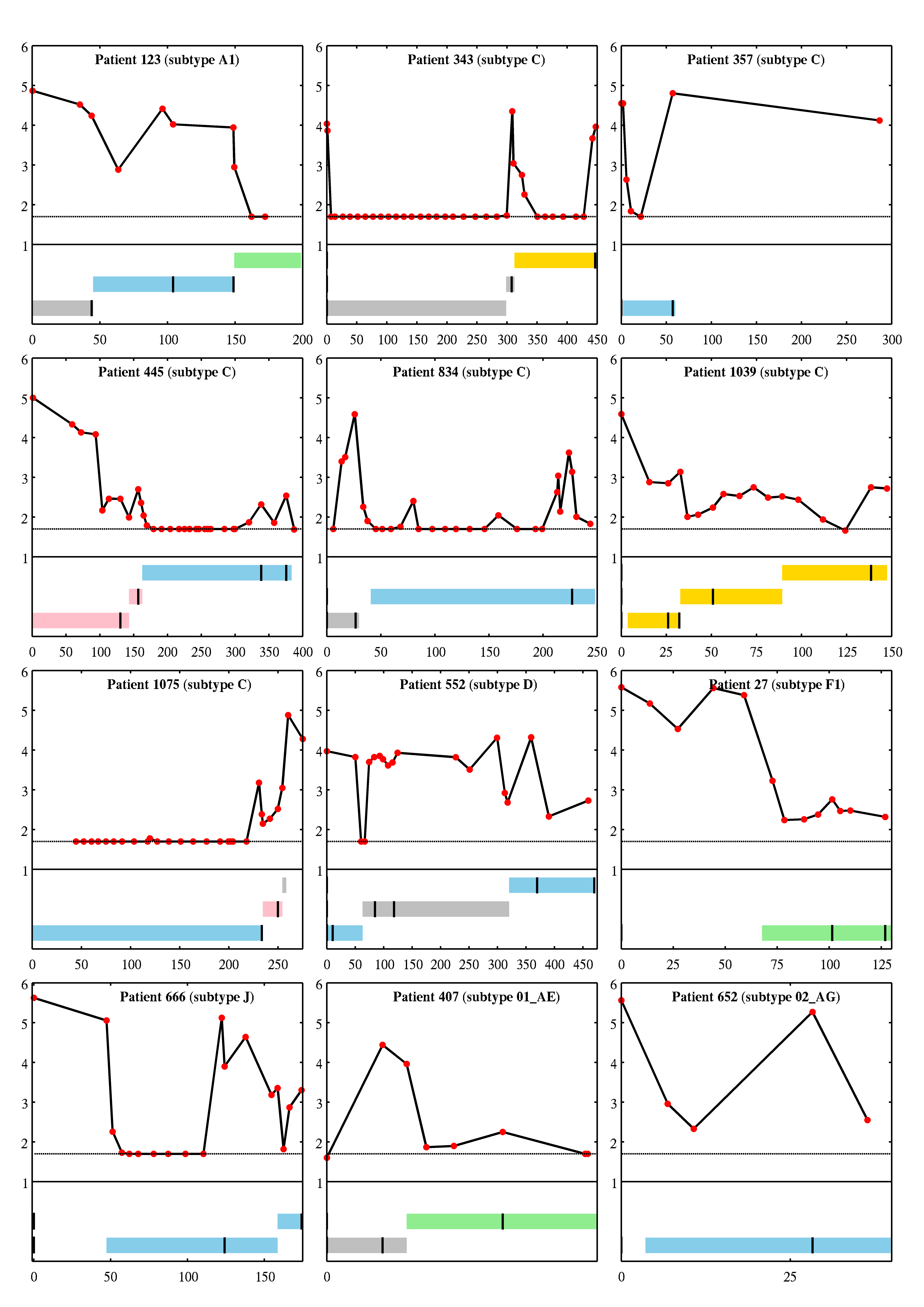

Supplement: Additional file 2: Software. — Toolbox S1: Our Matlab toolbox designed for visualizing longitudinal data of viral load, treatment period and sampling time. [file 12977_2014_79_MOESM2_ESM.zip › VisualizeLongitudinalData/LongtitudinalData.png]
